# Interaction of *Clostridioides difficile* infection with frailty and cognition in the elderly: a narrative review

**DOI:** 10.1186/s40001-023-01432-9

**Published:** 2023-10-17

**Authors:** Maria-Jose Fernandez-Cotarelo, Jasmine Y. Jackson-Akers, Stephanie E. Nagy-Agren, Cirle A. Warren

**Affiliations:** 1 Department of Internal Medicine, Hospital Universitario de Mostoles, Faculty of Health Sciences, Universidad Rey Juan Carlos, Calle Doctor Luis Montes S/N, Mostoles, 28935 Madrid, Spain; 2 División of Infectious Disease and International Health, University of Virginia, Charlottesville, VA USA; 3 Section of Infectious Diseases, Salem Veterans Affairs Medical Center, Virginia Tech Carilion School of Medicine, Roanoke, VA USA; 4 Division of Infectious Disease and International Health, University of Virginia, Charlottesville, VA USA

**Keywords:** *Clostridioides difficile* infection, Aging, Microbiome, Frailty, Cognition, Gut–brain axis

## Abstract

**Purpose:**

*Clostridioides difficile* infection (CDI) is the leading cause of antibiotic-related diarrhea and healthcare-associated infections, affecting in particular elderly patients and their global health. This review updates the understanding of this infection, with focus on cognitive impairment and frailty as both risk factors and consequence of CDI, summarizing recent knowledge and potential mechanisms to this interplay.

**Methods:**

A literature search was conducted including terms that would incorporate cognitive and functional impairment, aging, quality of life, morbidity and mortality with CDI, microbiome and the gut–brain axis.

**Results:**

Advanced age remains a critical risk for severe disease, recurrence, and mortality in CDI. Observational and quality of life studies show evidence of functional loss in older people after acute CDI. In turn, frailty and cognitive impairment are independent predictors of death following CDI. CDI has long-term impact in the elderly, leading to increased risk of readmissions and mortality even months after the acute event. Immune senescence and the aging microbiota are key in susceptibility to CDI, with factors including inflammation and exposure to luminal microbial products playing a role in the gut–brain axis.

**Conclusions:**

Frailty and poor health status are risk factors for CDI in the elderly. CDI affects quality of life, cognition and functionality, contributing to a decline in patient health over time and leading to early and late mortality. Narrative synthesis of the evidence suggests a framework for viewing the cycle of functional and cognitive decline in the elderly with CDI, impacting the gut–brain and gut–muscle axes.

## Case vignette

A 72-year-old woman is admitted to the hospital because of community-acquired pneumonia with respiratory failure, starting antibiotics and oxygen therapy. Her medical history includes hypertension, a benign thyroid nodule and mild cognitive impairment. Eight days after admission she is afebrile and the respiratory symptoms have improved, but she develops diarrhea and altered mental status. She is diagnosed with *Clostridioides difficile* infection and placed on oral Vancomycin. Should we be concerned about long-term impact of the infection on her functional and cognitive status?

## Background

Diarrhea is now a leading cause of mortality from infectious diseases in the US. While the overall mortality due to infections decreased from 42.95 to 34.10 deaths per 100,000 persons from 1980 to 2014, deaths from diarrheal diseases jumped from 0.41 to 2.41 per 100,000 persons [1]. This observation is likely driven by *Clostridioides difficile* infection (CDI) as it continues to be the most commonly known cause of antibiotic-associated diarrhea and healthcare-associated infections. The estimated number of incident CDI in the U.S. was 462,100, first recurrences was 69,800 and number of in-hospital deaths was 20,500 in 2017 [2]. Recent data from Europe report HA–CDI incidences ranging from 1.99 to 6.18 per 10,000 patient days, and CA–CDI incidences ranging from 0.56 to 1.4 per 10,000 patient days; the overall recurrence rate is 17% globally [3]. Although CDI also occurs in younger, lower-risk populations, advanced age remains the critical risk factor for severe disease, recurrence, and mortality. Persons > 65 years account for 72% of all CDI recurrence and 83% of all CDI deaths [4].

Although diarrhea is the most common symptom of CDI, older adults may present with additional atypical clinical features, such as acute confusion, altered mental status or other nonspecific symptoms of infection, including weakness and loss of physical functional capacity [5]. Altered mental status was the presenting symptom in one sixth of patients in a study, including cases from the community, hospitals and long-term care facilities (LTCF) [6]. Delirium was twice as frequent in hospitalized elderly with CDI as in controls in a two-center study [7].

Elderly patients with CDI are known to have higher rate of recurrence, more severe disease, poor response to treatment, and worse outcome [8–12]. With an increasing incidence, CDI has become an important condition not only in hospitals but also in LTCFs and in the community setting [9, 13–16]. *C. difficile* (CD) is acquired via fecal–oral transmission, and the intestinal microbiota plays an important role in defense against this infection. The gut microbiota composition changes with aging, leading to a reduction in the protective microbial diversity and a decrease in resistance to CD colonization [17, 18]. Dysbiosis, changes in gut physiology and function associated with aging, and the decline in the immune system contribute to put elderly people at risk for CDI [18, 19]. Unfortunately, aging as a risk factor is difficult to delineate from other features associated with the elderly, including frequent interactions with healthcare, hospitalizations, antibiotic exposure, comorbidities, polypharmacy, changes in microbiome and age-related changes in physiology and the immune response [9–11, 17, 19].

The authors conducted a narrative review of CDI in the elderly, with focus on its interaction with functional ability and cognition and its impact on overall health and mortality, resulting in a qualitative summary. The present study also presents what is known about potential mechanisms for this interplay.

## Methodology

A literature search in PubMed was conducted using the following search terms: (“clostridium difficile infection” OR “clostridioides difficile infection”) AND (aging OR elderly OR delirium OR “mental capacity” OR “mental competency” OR dementia OR cognition OR “cognition disorders” OR “cognitive impairment” OR “functional impairment” OR “functional status” OR “quality of life” OR “activities of daily living” OR frailty OR “frail elderly” OR delirium OR inflammation OR “inflamm aging” OR inflamm-aging OR immunosenescence OR neuroinflammation OR “brain–gut axis” OR “gut–muscle axis” OR “gastrointestinal microbiome” OR microbiota OR microbiome OR muscle OR skeletal). All articles published until June 2022 were considered.

## Main text

### Cognitive impairment and frailty as risk factors for CDI

A significant proportion of either frail or cognitively impaired elderly require medical or personal care in LTCF. About 1.3 million people in the US and 3.4 million people in European countries live in nursing homes (NH). Colonization with CD in this setting has been estimated to range from 5% to 51%, exceeding the rates in hospitalized patients [10, 14]. Transmission of CD occurs easily in healthcare facilities, since its spores contaminate and survive for long periods in the environment; thus, acquisition of the pathogen increases as does the length of stay [9]. Previous hospital admission poses a risk for CDI in both community-based and LTCF patients [9, 14, 20, 21]. One study that performed active sampling in hospitalized patients with CDI found highly mobile, more functional patients more likely to shed spores far from the bed, thus influencing environmental contamination [22]. Infection control measures are critical to avoid spreading of CD, but challenging to implement in LTCFs due to limitation of private spaces and trained staff compared to hospitals [9, 14]. Factors such as dementia or poor functional status of residents in LTCFs also affect the ability to implement measures to prevent transmission [14].

A retrospective study of CDI in LTCF residents found a significantly worse baseline status regarding activities of daily living in CDI cases compared to controls; it also showed higher overall comorbidity burden and drug utilization at baseline in CDI vs. non-CDI residents [20]. Another retrospective case–control study of CDI in NH residents utilizing the Monitored Dosage System from 200 pharmacies also found residents with CDI to have more functional impairment, with CDI acquired more frequently before entering the NH, mainly in a previous hospital admission [21]. CDI patients showed, however, a better cognitive baseline status in both studies. Impaired functional status has also been shown to be a risk factor for severe CDI among hospitalized older adults [23]. A two-center case–control study in the US and Europe among patients > 60 years found higher baseline functional debility and admission from NH or LTCF in patients with CDI compared to controls [7]. A retrospective study in Italy showed multimorbidity measured using Cumulative illness rating scale as a risk factor for CDI in hospitalized patients > 65 years [24].

Other studies suggest that functional debility, independent of age, is the critical risk factor for CDI. A prospective cohort study during an outbreak of CDI found debility with low Barthel scores and cognitive impairment with low Abbreviated Mental Test scores at the onset of symptoms highly associated with prolonged symptoms and more severe disease; there was not significant association between disease severity and age in this study [25]. Another study indicated that frailty and poor health status might be more important than age itself, as shown in a multivariable logistic regression using Medicare claims data that included comorbidities, health care utilization, and acute infections in the analysis [26].

### Impact of CDI on quality of life, cognition and functionality

Besides an increased risk for CDI, elderly patients have worse outcomes [9–12]. Consequences of CDI are the prolongation of hospital stay, a higher likelihood to be discharged to a NH, and a higher rate of readmissions in the subsequent months indicating overall health post-infection [27–29]. Thus, few studies have evaluated the impact of CDI on quality-of-life**.**

A multinational survey found patients with current or previous CDI scoring significantly worse in both mental and physical aspects compared to persons who had never experienced CDI, in all age groups, including > 65 years [30]. A French study described a drop in the state of health of patients with CDI in the short term, especially in women, patients with severe CDI and those older than 65 years, irrespective of their previous Charlson Comorbidity Index. These patients also experienced a quality-adjusted life year loss [31]. A disease-specific scale called “Cdiff32” demonstrated decreases in quality-of-life scores in physical, mental and social domains in patients with recurrent CDI [32]. A Canadian survey saw a reduction of self-assessed quality of life from prior to post CDI, with several respondents reporting to be unable to care for themselves, unable to work and/or needing some assistance with normal activities. Interestingly, the quality of life was lower when reported by a caregiver. A lasting impact after the resolution of CDI was also observed in a subgroup of patients in this study [33]. Extraintestinal chronic conditions such as arthritis and depression were reported to have worsened as a result of CDI in an observational cross-sectional study with an online survey [34].

Discharge to a NH or LTCF is a marker of functional loss following the infectious episode. Hospitalized patients developing CDI are more likely to be discharged to a LTCF [27, 29]. A nationwide retrospective study found significant association of discharge to NH with age > 65 years, comorbidities, severe CDI, and certain ribotypes [35]. A two-center study found elderly patients with CDI more likely to be discharged to NH or LTCF, experience functional decline or die during admission than matched controls [7]. A prospective study in a tertiary-care hospital analyzed the outcomes of CDI patients admitted from other facilities and discharged to LTCFs vs. controls discharged to home, showing longer hospital stay, a higher rate of readmission and 1-year mortality in cases [36].

### Morbidity and early and late mortality post-CDI

Mortality related to CDI is higher in the elderly and comorbid patient [13]. A systematic review that included 68 studies found older age as one of the most frequent risk factors for CDI recurrence, complicated disease, and mortality [8]. In addition to older age, many studies have also shown association of debility and poor baseline functional status with severity and mortality in CDI [21, 23, 25, 36, 37]. Admission from another acute hospital or a LTCF has also been described as an independent risk factor for CDI-related mortality in older adults [38]. Although with multifactorial etiology, delirium was associated with death within 30 days of diagnosis of CDI [7, 39]. In recurrent CDI, a case–control study in veterans identified cognitive dysfunction (OR 2.41), nutrition deficiency (OR 2.91) and to a lesser extent age (OR 1.04) as independent predictors of mortality, while the aggregate comorbidity burden was not [40]. More than half of the patients exhibited mental changes in a cohort of CDI cases requiring colectomy, being associated with higher mortality in these cases [41]. Frailty, measured using the validated Modified frailty index, also predicted mortality and prolonged hospital stay following colectomy for CDI [42]. A logistic regression model to predict mortality in geriatric patients suffering from CDI found pressure ulcers and malnutrition as main factors [43]. The Charlson comorbidity index in the group of deceased patients was higher than in the group of survivors, and there was no difference in mortality between the 65–84-year-old group and the > 85-year-old group in that study.

A case–control study including > 170.000 CDI cases in persons > 65 years analyzed the potential effect of CDI in morbidity, in addition to mortality [44]. Besides a higher all-cause 1-year mortality, CDI was associated with increased risk of 30-day, 90-day and 1-year hospitalization. Interestingly, when stratifying by risk factors for CDI and frailty indicators such as dementia or decubitus ulcers, the risk of mortality, transfer to a LTCF or skilled-nurse facility and subsequent hospitalization in this study was highest in persons with lowest probability of CDI. Indirect evidence of health impairment can be found in a study in NH residents that showed higher mortality, health care utilization and costs in the months after CDI compared to non-CDI controls [45].

Timing of mortality associated with CDI appears to be relevant in the elderly as well. A more advanced age, worse cognitive performance, and greater comorbidity burden per CCI were significantly associated with shorter time to mortality in LTCF residents with CDI [20]. A multicenter study in European hospitals found older age, cachexia, malignancy, Charlson Index and cognitive impairment as independent death predictors; older age, malignancy and cognitive impairment were also strongly correlated with shortening the time from CDI diagnosis to death [46].

Observational studies indicate that not only CDI is associated with early mortality (within 30 days of diagnosis) but surprisingly, also mortality at later timepoints, especially in the elderly. In our case-control study of CDI patients aged > 60 years conducted at 2 medical centers, including a local Veterans Hospital, we found that CDI diagnosis correlated with increased mortality even at 90 and 180 days (p = 0.004 and 0.011, respectively) post-diagnosis. Among those with CDI, a diagnosis of dementia was significantly associated with death at these later timepoints, and delirium was associated with functional decline or death [7]. Another study that included CDI patients > 80 years found long-term mortality associated with non-independent ADL baseline status [37]. These findings suggest that both early (occurring during hospitalization or 30-day post-diagnosis) and late (occurring > 30-day post-CDI) mortality are increased in the elderly indicating that CDI may contribute to a decline in patient function and health over time, ultimately leading to death. Interestingly, presence of some form of cognitive involvement or impairment appears to be particularly associated with mortality. Efforts to prevent CDI, therefore, should consider its short and long-term impact in elderly people with different burdens of diseases.

### Potential mechanisms underlying the vicious cycle of CDI in the elderly

**Fig. 1:**
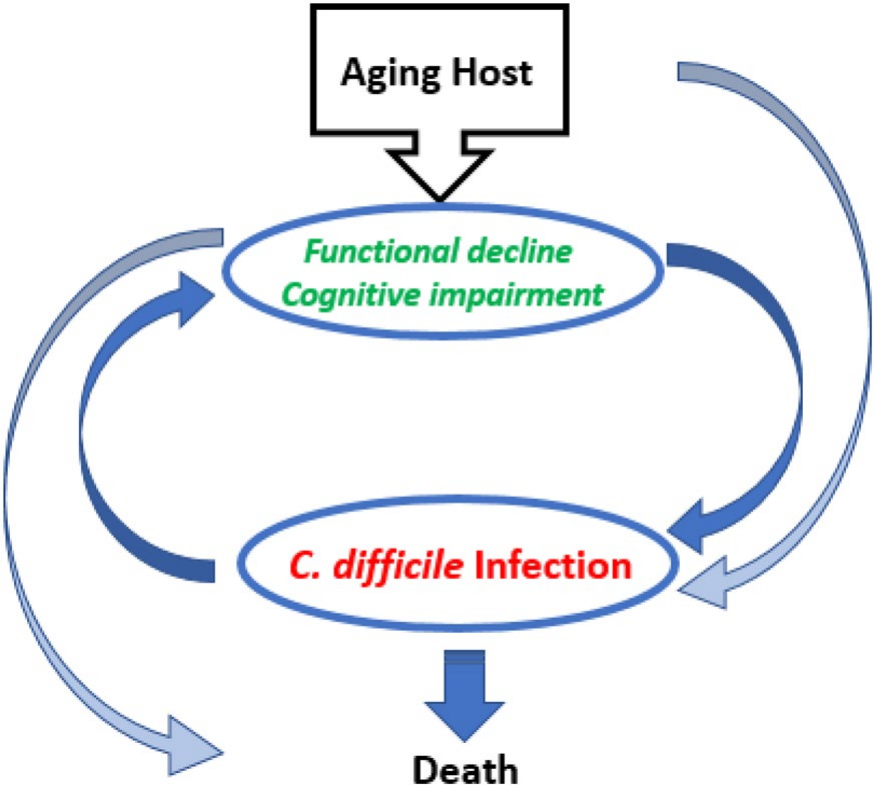
Onset of functional decline and cognitive impairment that can come with older age can increase susceptibility to CDI, while the infection, likewise, facilitates these debilities. Thus, a vicious cycle ensues that may eventually lead to death

The aging host is complex from both biological and non-biological standpoints. While the senescent immune system and changes in the microbiota increase susceptibility to infection and severe disease, comorbidities associated with aging increase risk of pharmacologic or surgical interventions that further dysbiosis and admission to healthcare facilities that increase exposure to CD spores. However, it appears that the onset of functional decline and cognitive impairment that can come with older age can increase susceptibility to CDI, while the infection, likewise, facilitates these debilities. Thus, a vicious cycle ensues that may eventually lead to death (Fig. 1).

Disruption of the intestinal microbiota is central to the pathogenesis of CDI. There has been increasing recognition of the importance of gut bacterial communities in maintaining overall health and the role of dysbiosis in the development of various diseases. With ageing, microbial diversity decreases which may lead to decline in intestinal epithelial barrier function. One of the underlying mechanisms of “inflamm-ageing” or “inflammaging” is the chronic, low grade systemic inflammation in response to exposure to luminal microbial products as a consequence of loss of epithelial integrity. Furthermore, this inflammatory process can be accelerated by CDI, even in mild disease. In a study of 36 patients, CDI results in a cascade of systemic cytokine production, including upregulation of IL-1B, IL-8, IL-16, and IL-17A, which are considered main cytokines mediating CD-associated disease [47].

Microbial infections lead to neurological damage by direct infection or by uncontrolled immune response [48]. Recent findings suggest the human microbiome can significantly affect brain function development and neuroinflammation. Neuroinflammatory responses are often a result of complex interaction between several chemokines, including chemokines (such as CCL2, CCL5 and CXCL1), cytokines (such as IL-6, and TNFα), and other inflammatory molecules, such as reactive oxygen species and prostaglandins [49]. It appears that the key cellular source of these factors are activated microglia, which are critical in maintaining synaptic connections and facilitating immunologic responses in the CNS [50]. Interestingly, neurological and neurodegenerative diseases can be influenced by peripheral factors, such as the gut microbiota. The microbial flora of the gastrointestinal tract can influence brain function by releasing neurotransmitters, hormones, and neuropeptides [51, 52]. Thus, further alterations in the microbiome caused by CDI might provoke inflammation-mediated blood–brain barrier breakdown facilitating development of neurodegenerative diseases [49].

More recently, interest in the gut–muscle axis has emerged. Several human studies have shown the association of microbiota composition with frailty [53]. Reduced microbiota diversity and decreased butyrate-producing bacteria were noted in elderly patients with diminished muscle mass [54]. Older adults with frailty had been shown to have predominance of fecal *Akkermansia*, *Parabacteroides*, and *Klebsiella* and depressed levels of *Faecalibacterium*, *Prevotella*, and other commensals, such as *Roseburia*, *Megamonas*, and *Blautia* [55]*.* Few studies have investigated the direct relationship of microbiota with muscle mass in animal models [56]. Compared to rats with normal muscle mass, rats with age-related sarcopenia have distinct microbiota composition and functionality, with alterations in protein, lipid and vitamin biosynthesis. Moreover, inflammatory biomarkers, such as IL-6 and white blood cells, were noted to be elevated in the presence of altered microbiome [54, 57]. Feeding of human commensal bacteria has been shown to inhibit muscle wasting and transfer of microbiota of high functioning older adults increased muscle strength in mice [58]. Similar to what likely occurs in the gut–brain axis, the persistent dysbiosis and inflammatory response induced by CDI may accelerate the catabolic process in the musculoskeletal system in the elderly.

### Research gaps

**Fig. 2:**
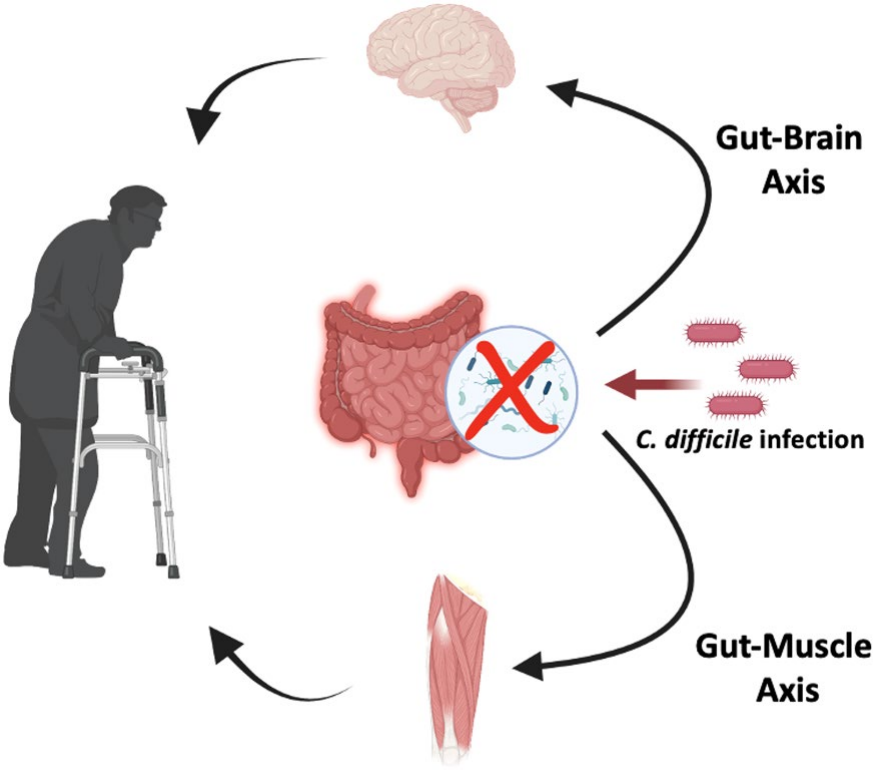
Gut–brain and gut–muscle axes in elderly likely influenced by *C. difficile* infection. CDI is facilitated by and sustains dysbiosis, induces intestinal inflammation and disrupts the epithelial barrier. Microbial products and inflammatory mediators gain access to the systemic circulation affecting extra-intestinal sites, such as the brain and musculoskeletal system. (Figure created using BioRender.com)

The risk factors for increased susceptibility to infection, severe disease and poor outcome, including impact on functional and cognitive abilities are numerous and intricately intertwined. It is important to consider these risks to ensure early diagnosis, avoid transmission, and appropriate treatment to minimize the burden of the disease in the elderly. We hypothesize that CDI in the elderly worsens dysbiosis-induced inflammation that facilitate both mental and musculoskeletal impairment (Fig. 2).

Although available clinical and research data suggest association of CDI with frailty, cognition and overall health in the elderly patient, significant gaps in knowledge exist. Functional and cognitive impairment need to be measured using validated tools to assess risk as well as determine the impact of CDI on the patient. It is known that survivors of severe sepsis are at high risk for cognitive impairment that lasts years after hospital discharge, and cognitive and functional impairment are seen in adults over age 50 who survive hospitalization for pneumonia [59–62]. In this regard, it is important that future research examine factors contributed by CDI alone vs. overall infectious status. Furthermore, patients with CDI need to be reassessed over time to detect early the impact of the disease on their general condition and test the benefit of microbiota-targeted intervention to quality of life, functional status, cognition and late mortality. Finally, given the complexity of the disease in the elderly, more mechanistic studies in aged animal models are needed to examine directly the interaction of the intestinal microbiota, inflammatory response, and brain and muscle functioning in the context of CDI.

### Data gathering limitations

Despite careful literature search, this narrative review might not have included some studies, due to availability or accessibility, especially those not published in English. In addition, the data interpretation may be influenced by the authors' subjectivity, bias, experience or expectations. Inherent to the nature of the narrative review, evidence-based responses to specific questions relating to potential cause and effect association of CDI with cognition and frailty are lacking and warrant further investigation.

## Conclusions

Advanced age remains a critical risk factor for severe CDI, recurrence, and mortality, although evidence suggests that frailty and poor health status pose risk approaching that of elderly age. CDI affects quality of life, cognition and functionality, contributing to a decline in patient health over time and leading to early and late mortality. The vicious cycle of CDI in the elderly facilitates dysbiosis and inflammatory response, impacting the gut–brain and the muscle–brain axes. Further research is needed to understand this interplay and improve patient outcomes.

## Data Availability

Data sharing is not applicable to this article as no data sets were generated or analysed during the current study.
